# Metabolism of barium in the human body after suicidal ingestion: A CARE-compliant case report

**DOI:** 10.1097/MD.0000000000030571

**Published:** 2022-09-16

**Authors:** Qiantong Zhang, Yuchao Wang, Xueyan Li, Zhoubo Wang, Heng Wang, Jianbo Yan

**Affiliations:** a Zhoushan Ambulance Center, Zhoushan, Zhejiang, China; b Zhoushan Municipal Center for Disease Control and Prevention, Zhoushan, Zhejiang, China; c Zhoushan Hospital, Zhoushan, Zhejiang, China.

**Keywords:** barium, human body, metabolism

## Abstract

Thus far, barium poisoning has been seldom reported and the metabolism of barium in human body has not been explored.

**Patient concerns:**

A 21-year-old young man was taken to the local hospital by “120 emergency medical services” after a suicidal attempt. About 100 mL of barium chloride solution with a concentration of 100 g/L was ingested, while the actual amount of ingested barium chloride solution was unclear because of immediate vomiting after the ingestion.

**Diagnoses:**

About 2 hours after the suicidal ingestion, the patient was presented with somnolence, the pulse rate was 67 beats per minute, the blood pressure was 158/92 mm Hg, but he exhibited no nausea or vomiting. About 3 hours after the ingestion, the blood concentration of potassium was 1.5 mmol/L.

**Interventions:**

The patient received gastric lavage by magnesium sulfate solution, intravenous sodium thiosulfate, and potassium supplementation. Other symptomatic treatments were applied simultaneously. To investigate the metabolism of barium in the human body, we measured the concentration of barium in 9 groups of paired serum and urine samples sequentially collected from the patient.

**Outcomes:**

The patient was rescued successfully.

**Lessons:**

The serum concentration of barium decreased rapidly in the first 24 hours. In this period, prompt and massive potassium supplementation and other symptomatic treatments are effective and recommended.

## 1. Introduction

Intentional or accidental barium ingestion is extremely rare, but it can cause death when the patient is not rescued timely, as reported on several occasions.^[1,2]^ Recently, a suicidal ingestion of barium chloride solution occurred in a medical facility and the patient was rescued successfully. We present the data for this case; for the first time, we measured the concentration of barium in 9 groups of paired serum and urine samples collected sequentially after ingestion, and revealed that barium can be metabolized rapidly during the first 24 hours.

## 2. Case report

A 21-year-old young male college student was taken to the local hospital by “120 emergency medical services” after he made a suicidal attempt. The ambulance was called by a college staff after approximately 100 mL of barium chloride solution had been ingested by the student. The concentration of the barium chloride solution, which he might have bought online, was 100 g/L. Due to immediate vomiting after the ingestion, the actual amount of barium chloride solution ingested was unclear. It was told by the college staff that the student had had minor depression for almost 1 year.

It was about 1 hour later when medical staff got to the patient. The patient was presented with abdominal pain and vomiting, his pulse rate was 73 beats per minute, his body temperature was 37°C and blood pressure was 110/70 mm Hg. Prior to transportation to the hospital, the patient was administered with oxygen inhalation and subjected to electrocardiographic monitoring.

Upon arriving at the emergency department of the local hospital about 2 hours after the suicidal ingestion, the patient was presented with somnolence, the pulse rate declined to 67 beats per minute, his blood pressure rose to 158/92 mm Hg, but he did not exhibit nausea or vomiting. The blood concentration of potassium was 2.74 mmol/L. Since the case was reported as barium poisoning, the patient received gastric lavage by magnesium sulfate solution, intravenous sodium thiosulfate, and potassium supplementation. Subsequently, the patient was transferred to ICU for further treatment. About 3 hours after the ingestion, the blood concentration of potassium dropped to 1.5 mmol/L. To accelerate the elimination of barium from the blood, blood dialysis was performed with sodium bicarbonate. About 0.5 hours after the start of blood dialysis, the blood concentration of potassium raised to 2.2 mmol/L. About 2.5 hours later, the blood dialysis was stopped. After continuous intravenous sodium thiosulfate and potassium supplementation for about 8 hours, the blood concentration of potassium returned to normal.

In order to clarify the pathogenesis mechanism, a group of paired samples, which contained 1 serum sample and 1 urine sample of the patient collected simultaneously, were sent to the local municipal center for disease control and prevention for barium analysis. The measurement was performed using an ICP-MS instrument (NexION 350D, Perkin Elmer, Waltham, MA, USA). It turned out that the concentration of barium in the serum and the urine was 4.3 mg/L and 3.9 mg/L, respectively. This group of paired samples were collected at about 9.5 hours after ingestion. Compared with serum and urine samples from the common population measured simultaneously (with both consistently below 0.01 mg/L), these values were considered extremely high. Meanwhile, the high concentration of barium in the urine was also good news indicating that barium was undergoing rapid metabolism. The high concentration of barium in the serum and urine sample of the patient helped clarify the pathogenesis of barium poisoning, and the treatment of the patient was assured.

**Figure 1.**
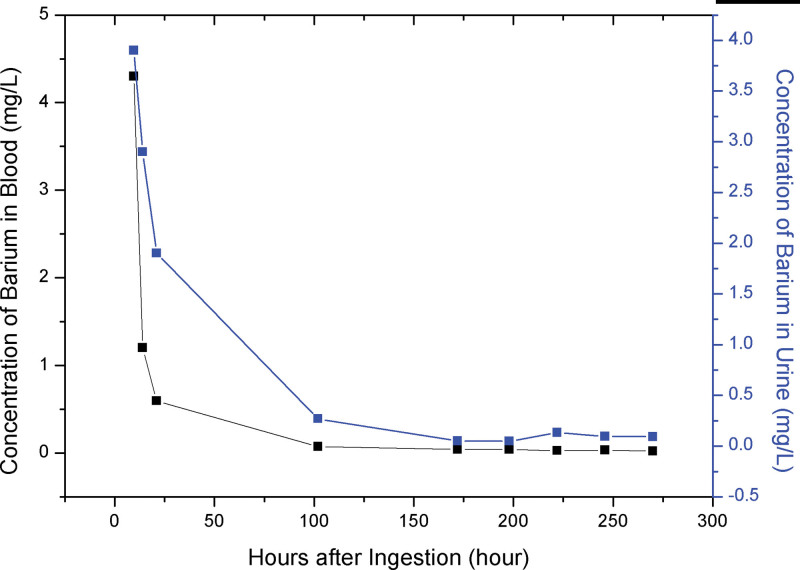
Concentration of barium in serum and urine after ingestion.

For further observations on the metabolism of barium in the human body, we measured the concentration of barium in another 8 groups of paired serum and urine samples collected sequentially from the patient (Fig. 1). The concentration of blood potassium returned back to normal about 14 hours after the ingestion. At this time point, the concentration of barium of the second group of paired serum and urine sample was 1.2 mg/L and 2.9 mg/L, respectively. In the third group of paired samples, collected about 21 hours after ingestion, the concentration of barium in the serum had dropped to 0.59 mg/L and to 1.9 mg/L in the urine sample, which were still high compared with the common population.

It was clear that the concentration of barium in the serum had decreased rapidly in the first 24 hours. Although the patient received blood dialysis, the high level of barium in the urine meant that barium in the blood was also being metabolized rapidly by the kidney. About 100 hours after the ingestion, the concentration of barium in the serum was <0.1 mg/L, while it was still 0.27 mg/L in the urine. Although it was a little higher than the concentration in the common population, the patient exhibited only slight acute symptoms. About 1 week later, the concentration of barium both in the serum and the urine was <0.05 mg/L, and the patient was recovered. From these results, we could infer that (1) it is the blood barium that causes the clinical symptoms, and the key to rescue the patient is to decrease this level, in addition to potassium supplementation; (2) the high level of barium in the urine is the result of the rapid metabolism of barium, and this high level may decline slower than that in the blood; (3) about 1.2 mg/L of barium in the blood may not cause hypokalemia. As for emergency medical treatment, potassium supplementation and blood dialysis as well as gastric lavage are effective measures.

## 3. Discussion

Acute barium poisoning has not been frequently reported, with a few cases in recent years.^[1,3,4]^ Since the first case of acute barium poisoning was published in 1984, hypokalemia has been accepted as the most profound symptom.^[5]^ In subsequent reports,^[3,4]^ potassium supplementation was recommended as essential treatment and the patients were rescued successfully. In addition, hemodialysis was used to promote clinical improvement.^[6]^ In the present case, potassium supplementation and hemodialysis were administered simultaneously, and the patient was rescued despite the severe hypokalemia (K ^+^ 1.5 mmol/L), which was near the lowest concentration ever reported. This highlights the utility of hemodialysis in the treatment of severe barium poisoning.

To the best of our knowledge, the current study is the first of its kind to report on the metabolism of barium in the human body. The results from 9 groups of paired serum and urine samples collected from the patient showed the rapid metabolism of barium in blood during the first 24 hours. This result was consistent with an earlier report, which simply mentioned that barium had disappeared from the patient’s blood within 24 hours after ingestion but provided no detailed data.^[7]^ Our results suggest that, in the first 24 hours after ingestion, prompt and massive potassium supplementation and other symptomatic treatments should be implemented. Treating the patient with hemodialysis may help facilitate the decrease of barium concentration in the blood.

## Author contributions

Conceptualization: Qiantong Zhang, Yuchao Wang, Heng Wang.

Writing – original draft: Qiantong Zhang, Xueyan Li.

Methodology: Yuchao Wang, Heng Wang.

Data curation: Qiantong Zhang, Zhoubo Wang, Xueyan Li, Heng Wang.

Writing – review and editing: Heng Wang, Jianbo Yan.

Supervision: Jianbo Yan.
